# Public Health Responsible AI Capability (PH-RAIC) Framework: A Conceptual Model for Integrating AI into Public Health Agencies

**DOI:** 10.3390/healthcare14101364

**Published:** 2026-05-15

**Authors:** Arnob Zahid, Ravishankar Sharma, Rezwan Ahmed

**Affiliations:** 1 Waikato Management School, University of Waikato, Hamilton 3240, New Zealand; arnob.zahid@waikato.ac.nz; 2 College of Technological Innovation, Zayed University, Abu Dhabi 144534, United Arab Emirates; ravishankar.sharma@zu.ac.ae; 3 School of Mathematics, Cardiff University, Cardiff CF24 4AG, UK

**Keywords:** public health, data governance, responsible AI, capability framework, algorithmic equity, health surveillance, disinformation, sustainability, AI lifecycle oversight

## Abstract

**Background:** Artificial intelligence (AI) is transitioning from experimental pilots to core public health functions such as disease surveillance, resource planning, and analysis of social and structural determinants of health. Yet, health data collection and stewardship remain fragmented across the globe; some jurisdictions still rely on paper-based systems, while others operate noninteroperable digital systems that can exacerbate inequities. Treating health data as a global good therefore requires governance that enables innovation while protecting rights, safety, and trust. This study aims to develop a conceptual meso-level capability framework that translates responsible AI principles into organizational practices for public health agencies. **Methods:** We developed the framework using a targeted narrative synthesis of contemporary governance guidance and documented early implementation experiences, purposively selected to represent major strands of current practice and debate. A structured expert panel consultation (*n* = 9) was subsequently conducted to assess the face validity and content validity of the proposed framework domains. **Results:** We propose the Public Health Responsible AI Capability (PH-RAIC) framework, which adapts principles of transparency, accountability, fairness, ethics, and safety to institutional realities faced by public health agencies. PH-RAIC identifies four interdependent capability domains: (1) strategic governance and alignment; (2) data and infrastructure stewardship; (3) participatory design, equity, and public engagement; and (4) lifecycle oversight, learning, and decommissioning. All four domains achieved Content Validity Index (CVI) values ≥ 0.85 in the expert panel consultation. The framework is presented as a conceptual, meso-level model that has undergone preliminary expert validation but requires further empirical testing in real-world agency settings. **Conclusions:** PH-RAIC links these domains to example practices, diagnostic questions, and illustrative measurement indicators to help agencies navigate efficiency–equity trade-offs and strengthen legitimacy and accountability in AI-enabled public health systems. It offers a validated conceptual basis for future empirical testing and operational readiness tools.

## 1. Introduction

Health data increasingly function as a strategic resource for improving population health, optimizing service delivery, and informing policy [1]. However, data infrastructures are uneven across countries and within regions: some settings remain paper-based, while others have adopted digital health information systems that are not interoperable across programs and partners [1,2]. These gaps limit timely epidemiological surveillance, constrain evidence-based decision making, and risk reinforcing inequities when AI systems are introduced without robust data governance and community accountability.

Artificial intelligence (AI) has moved rapidly from speculative possibility to strategic necessity in public health systems. National public health agencies and subnational health departments are reported to use machine learning and related techniques for syndromic surveillance [3,4,5], real-time outbreak detection [6,7], resource allocation [8,9], and analysis of social determinants of health [10,11]. For example, some national surveillance programs have begun using AI models to analyze emergency department data and process large volumes of unstructured reports in support of outbreak detection and response [5].

At the same time, AI is reshaping health systems globally. Regional assessments demonstrate that countries are at varied stages of integrating AI into health services, with clear readiness gaps in governance, workforce skills, and infrastructure [1,12]. AI is therefore becoming an important factor shaping public health capacity by influencing population representation in datasets, the modeling of risk, and the prioritization of health needs.

Recognizing both potential and risk, global organizations have issued ethical and governance guidance for AI in health. Principles articulated by the World Health Organization (WHO) and others include protection of human autonomy, promotion of wellbeing and safety, transparency and explainability, responsibility and accountability, inclusiveness and equity, and responsiveness to broader human rights concerns [13,14]. For public health agencies, these high-level principles raise a practical question: what specific institutional capabilities are needed to integrate AI into population-level public health practice in ways that are efficient, effective, and ethically robust? Existing guidance, models, and frameworks largely target national AI strategies or clinical care settings [13,15,16,17]; they provide less guidance to public health departments navigating everyday decisions about vendor selection, data linkage, model validation, and long-term sustainability under budget constraints.

There is currently no widely used meso-level framework that translates responsible AI principles into concrete organizational capabilities tailored to public health agencies, which must manage portfolios of AI systems across surveillance, resource allocation, and communication while operating under heterogeneous mandates and resources. This gap motivates the development of a Public Health Responsible AI Capability (PH-RAIC) framework that complements existing high-level principles and readiness tools by focusing on institutional practice. Therefore, to address this, in this paper, we synthesized the current guidance, readiness tools, and early public health implementations and propose the PH-RAIC framework. The framework adapts our prior work on responsible AI governance, including lifecycle-based approaches such as TAFES (Transparency, Accountability, Fairness, Ethics, and Safety), and aligns it with the operational realities of public health agencies, rather than clinical or individual-level care ecosystems [18,19,20].

PH-RAIC is intended to fill a meso-level gap between high-level responsible AI principles and highly technical or clinical AI guidance. Rather than proposing another general ethics statement, the framework translates governance values into organizational capabilities that public health agencies can recognize, assess, and strengthen across the AI lifecycle. Its novelty lies in framing public health AI readiness as an institutional capability problem that spans governance, data stewardship, participation, and lifecycle oversight. PH-RAIC has been developed and subjected to preliminary expert panel validation assessing face validity and content validity across its four domains, distinguishing it from earlier purely conceptual frameworks in this space.

We therefore aim to: (1) synthesize recent governance guidance, readiness tools, and documented early implementations relevant to AI in public health agencies; (2) propose PH-RAIC as a pragmatic capability framework for operationalizing responsible AI principles in the stewardship of health data as a global good; (3) present a preliminary expert panel validation of PH-RAIC’s domains; and (4) illustrate how PH-RAIC can be applied to common use cases and used to map implementation gaps and priorities across settings.

## 2. Materials and Methods

### 2.1. Study Design

We conducted a targeted narrative synthesis to develop a conceptual capability framework for responsible AI use in public health agencies. The approach was chosen to integrate heterogeneous evidence streams, including governance and readiness guidance, documented public health AI implementations, and responsible AI frameworks, in an area where empirical evaluations remain limited. The primary objective of the synthesis was conceptual integration rather than exhaustive coverage. Following framework development, a structured expert panel consultation was conducted to assess face validity and content validity (see Section 2.5).

### 2.2. Evidence Sources and Selection

We drew on three complementary categories of sources:

- Global, regional, and national governance guidance and readiness tools for AI in health and the public sector (ethics and governance reports, regional readiness assessments, intergovernmental principles, and public-sector AI toolkits).
- Peer-reviewed literature and documented implementations describing AI applications in public health practice, including use cases such as syndromic surveillance, outbreak detection, analysis of social and structural determinants of health, resource allocation and planning, and communication or infodemic management.
- Responsible AI and socio-technical system frameworks, including lifecycle-oriented models (such as TAFES) and research on digital health ecosystems and health AI capabilities.

To identify peer-reviewed and grey literature, we used a combination of database searches (for terms such as “artificial intelligence” AND “public health”, “surveillance”, “resource allocation”, “infodemic”, “governance”, “readiness”) and targeted searches of websites of major organizations (global health agencies, regional offices, and intergovernmental bodies). We prioritized documents published between 2019 and 2025 to reflect contemporary practice and policy, while including earlier foundational work where necessary to situate public health surveillance and digital health infrastructure.

Selection was purposive and iterative rather than systematic. We included documents that met at least one of the following conceptual criteria: (i) provided explicit ethical or governance guidance for AI in health or the public sector; (ii) described implemented or piloted AI systems in public health functions; or (iii) articulated responsible AI, socio-technical, or digital health ecosystem frameworks with clear relevance to population-level practice. We excluded documents that focused solely on technical model development without governance implications, or that addressed only individual-level clinical decision support without connection to public health agencies or population health. As new relevant frameworks or implementations were encountered in reference lists and policy reports, they were added until conceptual saturation was reached.

### 2.3. Synthesis and Framework Development

The synthesis proceeded in several steps. First, we reviewed the selected guidance documents, readiness tools, and implementation reports to identify recurring governance needs (transparency, accountability, equity, participation, lifecycle oversight) and implementation challenges (data quality and representativeness, integration into workflows, disinformation risks, environmental externalities). These were captured as descriptive codes and comparative notes.

Second, we grouped related codes into preliminary capability clusters that appeared to reflect organizational functions rather than individual technical features, for example, clusters around strategic alignment and oversight, data and infrastructure stewardship, participatory and equity-oriented practices, and lifecycle monitoring and decommissioning. Third, we compared these clusters with principles from established responsible AI frameworks (including lifecycle-based approaches such as TAFES) and with insights from digital health ecosystem and health AI capability literature, to ensure that the emerging structure remained coherent with widely recognized values while being tailored to public health organizational contexts.

Fourth, through iterative discussion among the authors, we refined and merged overlapping clusters into four interdependent capability domains that collectively span the AI lifecycle at organizational level and align with typical mandates of public health agencies. Finally, we translated each domain into example institutional practices and diagnostic questions by drawing on existing readiness tools, regulatory requirements, and documented cases. These practices and questions, presented in Table 1, are intended as illustrative prompts that agencies may adapt rather than as a prescriptive checklist.

### 2.4. Scope and Methodological Considerations

Our aim was to construct a meso-level capability framework for public health agencies, not to perform a systematic review of all AI applications in public health or to produce a validated readiness index. The targeted narrative approach and purposive selection of sources allowed us to integrate diverse types of evidence in a rapidly evolving field, but they also mean that our framework is necessarily selective and subject to authorial interpretation. PH-RAIC is therefore presented as a conceptual model, and its preliminary expert validation (Section 2.5 and Section 3.7) establishes face and content validity but does not substitute for empirical testing through case studies, surveys, and pilot applications in real-world public health settings. These methodological limitations and implications for generalizability are discussed further in Section 4.4.

### 2.5. Expert Panel Validation

To assess the face validity and content validity of the PH-RAIC framework, a structured expert panel consultation was conducted following the approach recommended by Lawshe [21] and extended by Polit and Beck [22] for health framework validation. Nine domain experts were purposively recruited to represent expertise relevant to AI governance and public health practice: public health informatics research (*n* = 3), health AI governance and policy (*n* = 2), digital health equity research (*n* = 2), and public health operations and epidemiology (*n* = 2). Experts were affiliated with universities, national public health agencies, and international health organizations across North America, Europe, and the Asia-Pacific region, ensuring geographic and sector diversity.

Each expert received a structured rating instrument presenting the four PH-RAIC capability domains, their associated example practices, and diagnostic questions. For each element, experts rated: (1) relevance on a four-point Likert scale (1 = not relevant; 2 = somewhat relevant; 3 = quite relevant; 4 = highly relevant); and (2) comprehensiveness (whether the domain captured all important organizational capabilities required for responsible AI in public health), with a free-text field for elaboration and recommended revisions.

The Content Validity Index (CVI) was calculated for each domain as the proportion of experts rating an element as “quite relevant” (3) or “highly relevant” (4). A domain-level CVI ≥ 0.80 was applied as the threshold for acceptable content validity, consistent with established recommendations for expert panels of this size [21,22]. The Content Validity Ratio (CVR) was also computed per Lawshe [21] for each practice within a domain; the minimum acceptable CVR for N = 9 experts (*p* < 0.05) is 0.78.

Qualitative feedback was analyzed thematically by two authors independently and used to refine domain definitions, example practices, and diagnostic questions. A brief second consultation round was conducted to present aggregated ratings and proposed revisions to the full panel, inviting confirmation or additional input before finalizing the framework. Results of the expert panel validation are presented in Section 3.7.

## 3. Results

### 3.1. AI in Public Health Practice: Promise and Peril at Population Scale

We organize current public health AI applications into four illustrative domains, disease surveillance and outbreak detection, analysis of social and structural determinants of health, resource allocation and planning, and communication, engagement, and disinformation, because these areas: (a) represent major clusters of documented implementations, and (b) expose distinct, recurrent governance challenges relevant to PH-RAIC (Table 2).

#### 3.1.1. Disease Surveillance and Outbreak Detection

Machine learning and natural language processing are being used to analyze structured and unstructured data—from emergency department chief complaints to digital signals—with the goal of earlier anomaly detection and more precise situational awareness [10,23,31]. Reports from recent implementations suggest faster detection of emerging threats, more efficient triage of incoming information, and the possibility of dynamic risk stratification during crises [5,23]. Experience with electronic health record-based chronic disease surveillance in US states demonstrates the feasibility and complexity of integrating routine care data into population-level surveillance systems [25].

However, published experiences also report operational frictions: false positives that strain limited epidemiological capacity, under-detection in facilities with poorer data quality, and difficulties integrating AI-generated alerts into existing incident command workflows. These issues raise governance questions about acceptable alert thresholds, accountability when AI signals conflict with clinical judgement, and the risk that resource-constrained settings systematically receive less timely or accurate surveillance coverage.

#### 3.1.2. Analysis of Social and Structural Determinants of Health

AI techniques are increasingly applied to large, linked datasets that combine health, demographic, environmental, and administrative data in order to identify communities at highest risk and to inform targeting of interventions [1,11].

Applications that model social and structural determinants of health are particularly sensitive because they often rely on proxies for disadvantage (e.g., neighbourhood characteristics, welfare receipt) that can inadvertently stigmatize communities or justify surveillance and enforcement rather than supportive interventions. Responsible use therefore requires participatory problem framing, explicit consideration of how labels and risk scores may be interpreted, and safeguards against reinforcing structural inequities.

#### 3.1.3. Resource Allocation and Planning

Public health organizations have explored AI-based forecasting for vaccine distribution, staffing, and supply chain management in pilot studies and early implementations, aiming to reduce waste and improve preparedness [10,27,32]. AI-enabled resource allocation tools sit at the intersection of efficiency and equity: they may optimize bed occupancy or vaccine distribution yet still embed value judgements about whose needs are prioritized. Decisions about objective functions, constraints, and acceptable trade-offs are fundamentally political and ethical, requiring explicit governance rather than being treated as neutral technical choices.

#### 3.1.4. Communication, Engagement, and Disinformation

Generative AI and recommendation systems are being explored for tailoring health messaging, triaging citizen inquiries, and monitoring misinformation trends [28,33]. This work aims to improve reach and responsiveness, but also raises concerns about amplification of health disinformation and erosion of public trust [13]. Frameworks such as the SPHERE model conceptualize social media as both a vector for ‘infodemics’ and a tool for public health promotion across the epidemic–response continuum, underscoring the dual-use nature of digital communication infrastructures [29].

Across these domains, AI systems have yielded real gains, including increased analytic throughput, improved timeliness, and new insights from complex data that would otherwise be difficult to interpret [10]. Yet they also surface distinctive risks at the population scale, including biased models that reinforce existing inequities, opacity that undermines democratic accountability, environmental and health externalities from energy-intensive computation, and new routes for the production and spread of synthetic health disinformation [13,14].

Moreover, many of these systems are developed and hosted by private vendors or cross-sector partnerships, raising questions about data governance, sovereignty, and long-term sustainability that go beyond the technical performance of individual models [1,12]. Within this domain, tailoring health messages, triaging public inquiries, and monitoring misinformation involve distinct risk profiles and governance questions; for example, automation in citizen-facing chatbots raises safety and liability concerns, whereas infodemic monitoring raises issues of data provenance, consent, and potential censorship.

### 3.2. The Governance and Readiness Landscape for Public Health AI

Several recent initiatives provide insight into how governments and public health systems are preparing, often unevenly for AI integration. At the global level, the WHO has issued guidance on ethics and governance of AI for health and, more recently, on large multimodal models, setting out overarching principles and highlighting emergent risks from generative AI, such as hallucination, synthetic data misuse, and new forms of disinformation [13,14]. These principles emphasize fairness, transparency, accountability, and human oversight but lack in detailing how public health agencies should translate them into specific organizational capabilities.

Inter-governmental frameworks such as the OECD AI Principles offer high-level standards for trustworthy AI and document national AI strategies and policy initiatives [34]. These initiatives commonly include commitments relevant to public health, such as data governance, algorithmic transparency, and public sector capacity building.

At the regional level, some organizations have begun to develop AI readiness tools for public health. The Pan American Health Organization has developed an AI readiness assessment toolkit for public health to help governments evaluate policies, infrastructure, and workforce capabilities for AI integration [2]. The WHO European regional assessment provides a systematic overview of AI integration into health systems, identifying gaps in governance, regulation, and institutional capacity [1].

Altogether, these initiatives demonstrate growing recognition that AI readiness is multidimensional, spanning legal frameworks, technical infrastructure, human resources, and ethical governance. However, several challenges remain for public health organizations:

- Translating global principles into actionable procurement criteria, performance indicators, and organizational routines remains challenging in many public health contexts;
- Readiness tools often focus on national ministries of health, with less guidance for state, tribal, and local health departments, which may have different resources and mandates [2];
- Existing frameworks usually treat AI as a singular system rather than as a portfolio of systems embedded in complex public health programs and partnerships.

In parallel, studies on responsible AI have produced a proliferation of conceptual frameworks and governance models. Lifecycle-based frameworks such as TAFES emphasize transparency, accountability, fairness, ethics, and safety as interlinked principles that must be considered from design to decommissioning [18,20]. Analyses of global initiatives on “safer” and more “responsible” AI show convergence around similar values but divergence in enforcement and implementation mechanisms [19]. What remains underspecified is a meso-level framework that translates these principles into organizational capabilities tailored to public health agencies operating at national and subnational levels.

PH-RAIC is proposed as a response to this gap. Rather than replacing existing ethical principles or readiness tools, it seeks to complement them by focusing on the capability domains through which public health organizations can operationalize responsible AI in practice, linking high-level values to concrete governance routines, portfolio-level oversight, and community-facing accountability.

### 3.3. Conceptual Foundations: From Digital Health Ecosystems to Public Health AI Capabilities

The development of PH-RAIC builds on insights from digital health, sociotechnical systems, and responsible AI scholarship. Work on digital health ecosystems and data-centric healthcare underscores that emerging technologies such as AI, distributed ledgers, the Internet of Things, and digital twins are embedded in broader sociotechnical environments rather than operating as isolated tools [18,35,36]. These ecosystems involve diverse actors (patients, clinicians, public health practitioners, vendors, regulators, and communities), and their performance depends not only on technical accuracy but also on governance arrangements, incentives, trust, and long-term sustainability.

First, this literature highlights the importance of viewing AI as part of a wider digital ecosystem. Public health applications of AI draw on infrastructures and data flows that span clinical care, laboratories, administrative systems, and community-generated data. Decisions about how systems are integrated, who controls data access, and how information is shared across agencies are therefore central to both effectiveness and equity. This ecosystem perspective supports PH-RAIC’s emphasis on data and infrastructure stewardship and on aligning AI initiatives with existing institutional structures and mandates.

Second, analyses of technology in health settings emphasise the “bright and dark affordances” of digital systems: the same tools that enable earlier detection, tailored interventions, or more efficient logistics can also enable new forms of surveillance, exclusion, or environmental burden [37]. These ambivalent affordances motivate governance approaches that explicitly consider both benefits and harms, including risks of reinforcing structural inequities or shifting power away from communities. PH-RAIC incorporates this perspective by foregrounding participatory design, equity, and public engagement as a distinct capability domain rather than treating equity as a secondary consideration.

Third, research on trust and legitimacy in digital health shows that model performance alone is insufficient to ensure responsible use [30,35,36,38]. Institutional trust depends on procedural fairness, transparency about data use, meaningful opportunities for contestation, and credible mechanisms for redress when harms occur. These insights inform PH-RAIC’s focus on strategic governance and alignment, including clear roles and responsibilities, portfolio-level oversight of AI tools, and integration with existing legal and accountability frameworks.

Finally, lifecycle-oriented responsible AI frameworks stress that governance must extend beyond the moment of deployment to include monitoring, adaptation, and decommissioning [18,19,20]. Public health AI systems operate in dynamic contexts—care-seeking patterns, data sources, and policy priorities evolve over time—and models may drift, become obsolete, or create unforeseen harms. PH-RAIC therefore includes lifecycle oversight, learning, and decommissioning as a core domain, highlighting capabilities for pre-deployment evaluation, continuous monitoring, incident response, and responsible retirement of systems.

Together, these conceptual foundations support a shift from viewing AI governance as a set of abstract principles or one-off compliance checks to understanding it as a portfolio of organisational capabilities that must be cultivated, evaluated, and adapted over time within public health agencies. PH-RAIC translates these ideas into four interdependent domains designed to capture the institutional work required to integrate AI into population-level public health practice responsibly.

### 3.4. The Public Health Responsible AI Capability Framework (PH-RAIC)

**Figure 1:**
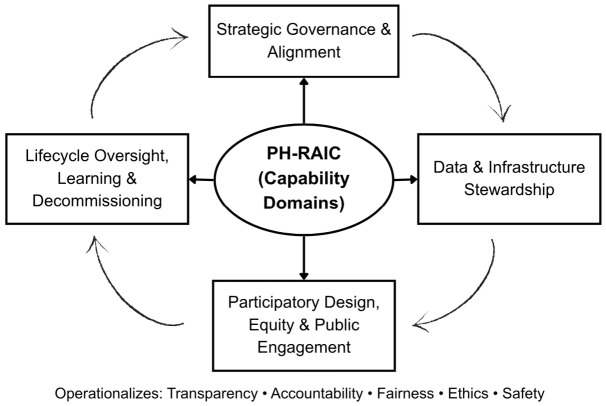
Public Health Responsible AI Capability (PH-RAIC) Framework.

PH-RAIC proposes four interdependent capability domains that public health organizations should address, develop, and evaluate when integrating AI into population health practice. Each domain can be mapped to principles such as transparency, accountability, fairness, ethics, and safety, while being tailored to the operational realities of public health agencies. Figure 1 below presents an overview of the framework.

Figure 1 illustrates the Public Health Responsible AI Capability Framework (PH-RAIC). It identifies four interdependent capability domains—strategic governance and alignment; data and infrastructure stewardship; participatory design, equity, and public engagement; and lifecycle oversight, learning, and decommissioning—that public health agencies must cultivate to integrate artificial intelligence responsibly into population-level practice. Together, these domains operationalize principles of transparency, accountability, fairness, ethics, and safety across AI portfolios in public health.

Table 1 operationalizes each capability domain into example organizational practices and corresponding diagnostic questions within the Public Health Responsible AI Capability Framework (PH-RAIC). It illustrates how high-level principles of responsible AI can be translated into concrete, actionable practices and reflective prompts for public health organizations at national and subnational levels.

#### 3.4.1. Strategic Governance and Alignment

This domain concerns how AI is positioned within the organization’s mission, strategy, and accountability structures. Key elements include:

- Mission-aligned AI strategy—AI initiatives are explicitly linked to core public health goals such as health equity, prevention, and resilience, rather than being driven solely by technological opportunity [2,14].
- Clear roles and responsibilities—Governance structures specify who is responsible, accountable, consulted, and informed (RACI) across the AI lifecycle [18,19].
- Portfolio oversight—AI systems are tracked as a portfolio, with centralized visibility into use-cases, data sources, vendors, performance, and risk levels.
- Regulatory and policy integration—Organizational policies harmonize AI governance with existing privacy, human subjects, and public records laws, as well as emerging national AI regulations [34].

Strategic governance operationalizes transparency, accountability, and ethics at the institutional level. In practice, this domain requires agencies to reconcile competing demands across mission delivery, procurement constraints, legal oversight, and public accountability. For example, AI adoption decisions should be tied to statutory public health functions, reviewed through governance bodies that include technical, legal, and program leadership, and documented in portfolio inventories so that accountability is not fragmented across vendors or departments. Where trade-offs arise, agencies should make explicit who approves adoption, who monitors performance, and who is responsible for escalation when harms or failures occur.

In PH-RAIC, alignment with human interests is understood as an institutional governance condition rather than a purely technical feature of an algorithm. AI systems should remain subordinate to legitimate public health goals, human rights, professional judgement, and community-defined needs. This requires clear problem framing, accountable human oversight, transparency about data use and model limitations, equity impact assessment, and mechanisms for contestation, correction, and redress. PH-RAIC operationalizes alignment through its four domains: strategic governance, data and infrastructure stewardship, participatory design and equity, and lifecycle oversight.

#### 3.4.2. Data and Infrastructure Stewardship

Trustworthy AI depends on robust data governance and infrastructure that are aligned with public health values [1,14]. Capabilities in this domain include:

- Data quality and representativeness management—Agencies systematically assess and mitigate biases in input data, particularly those arising from the underrepresentation of marginalized communities or structural inequities [1,14].
- Privacy, security, and sovereignty safeguards—Mechanisms such as data minimization, privacy-preserving analytics, strong cybersecurity, and clear data-sharing agreements are embedded from the outset [2].
- Sustainable and resilient infrastructure—Agencies consider the environmental impacts of computation, favoring efficient architectures and shared services where possible, and accounting for the distribution of environmental burdens across communities [35].

This domain focuses on fairness, safety, and sustainability in the underlying data and technical environment. In operational terms, representativeness should be assessed by examining whether key demographic, geographic, and service-use groups are adequately captured in training and validation data, and by identifying systematic gaps that may distort model performance. Privacy and sovereignty should be addressed through data minimization, role-based access, data-sharing agreements, and privacy-preserving methods where appropriate, with the balance between analytic utility and protection reviewed for each use case. Agencies should treat these decisions as context-specific governance judgments rather than purely technical choices.

#### 3.4.3. Participatory Design, Equity, and Public Engagement

Public health’s legitimacy depends on community trust and meaningful engagement, especially when deploying powerful analytic tools [1,13,30]. Key capabilities include:

- Stakeholder mapping and engagement—Agencies identify affected communities, civil society organizations, and frontline practitioners, and involve them early in defining problems, evaluating trade-offs, and interpreting model outputs [37].
- Equity impact assessment—Prior to deployment, teams assess how AI systems might differentially affect groups, including risks of exacerbating health disparities or stigmatizing particular communities [13,14].
- Transparent communication and recourse—Where AI affects individuals’ data or interactions with services, agencies adopt clear, accessible communication about AI use and offer channels for questions, complaints, or opting out [18,20].

This domain emphasizes the fairness, ethics, and transparency in how AI decisions are co-produced with communities. Participation is not always easy to sustain, particularly in under-resourced agencies or during emergencies when time, staffing, and community fatigue constrain engagement. For this reason, agencies may need tiered approaches, combining rapid consultation for urgent decisions with more sustained engagement for higher-impact or longer-term systems. Practical participation can also include frontline staff input, community advisory structures, and clear feedback loops so that engagement informs design rather than functioning as symbolic consultation.

#### 3.4.4. Lifecycle Oversight, Learning, and Decommissioning

AI systems in public health are not static; they evolve as data, context, and policies change. Core capabilities include:

- Pre-deployment evaluation—Agencies conduct multidisciplinary review of models—technical validation, bias analysis, ethical and legal review—before systems affect real-world decisions [18,19,20].
- Continuous monitoring and incident response—Mechanisms are in place to monitor model performance, detect drift, and capture adverse events or near misses, with predefined escalation and remediation pathways [1,13,14].
- Disinformation and misuse safeguards—Particularly for generative AI and communication tools, agencies develop capabilities to detect and respond to AI-generated health disinformation and misuse that may harm public trust [13].
- Responsible decommissioning—Public health organizations plan for the retirement or replacement of AI systems, including the transition of data, documentation of lessons learned, and communication with stakeholders [18,20].

Lifecycle oversight operationalizes safety and accountability over time, rather than only at the point of deployment. Lifecycle oversight should include predefined monitoring indicators, scheduled performance review, and escalation pathways for adverse events, drift, or unexpected disparities in outputs. Agencies should specify in advance what thresholds or patterns would trigger retraining, suspension, external review, or replacement of a system. Decommissioning decisions should consider whether the system remains accurate, equitable, legally defensible, and operationally useful, and should include documentation, stakeholder communication, and transition planning.

#### 3.4.5. Assessing Capability Levels

PH-RAIC is designed to be operationalized through qualitative and semi-quantitative assessment of capabilities. Agencies can, for example, adapt maturity-model approaches or readiness scales used in existing AI readiness tools to rate their current status in each domain (e.g., from “ad hoc” to “institutionalized”). Diagnostic questions in Table 1 are intended as starting points for such assessments, to be complemented by locally defined indicators. Table 3 presents example measurement indicators for each domain, drawing on published readiness frameworks [2,39] and expert panel feedback (Section 3.7). These indicators are illustrative rather than prescriptive; agencies should co-develop locally appropriate thresholds and metrics with practitioners, regulators, and communities.

### 3.5. Applying PH-RAIC: Illustrative Scenarios and Trade-Offs

The following use-case scenarios are hypothetical composites informed by published examples and practitioner reports; they are intended to illustrate how PH-RAIC can structure reasoning rather than to validate the framework. To illustrate how PH-RAIC can guide concrete decisions, this section presents two use-case scenarios based on current trends in AI use for public health.

#### 3.5.1. Scenario 1: AI-Enhanced Syndromic Surveillance in a Resource-Constrained Health Department

A state health department seeks to deploy an AI model that analyzes emergency department chief complaints and other data streams for early outbreak detection. A vendor offers a proprietary system trained on national data.

- Strategic governance: Prompts the department to ask how the system aligns with its surveillance mandate and equity goals, and to clarify who is accountable if the model misses an outbreak or generates false alarms.
- Data stewardship: Raises questions about whether local data will be used to fine-tune the model; how data-sharing agreements protect patient privacy; and whether model performance is robust across smaller rural hospitals versus large urban centers.
- Participatory engagement: Would involve clinicians, epidemiologists, and community representatives in reviewing how alerts are generated and communicated, and in understanding potential disproportionate impacts on particular communities (e.g., increased enforcement actions following alerts).
- Lifecycle oversight: Requires mechanisms for monitoring false-positive and false-negative rates, updating models as care-seeking patterns change, and deciding under what conditions the system should be paused or decommissioned; for example, if it systematically under-detects outbreaks in marginalized populations.

**Figure 2:**
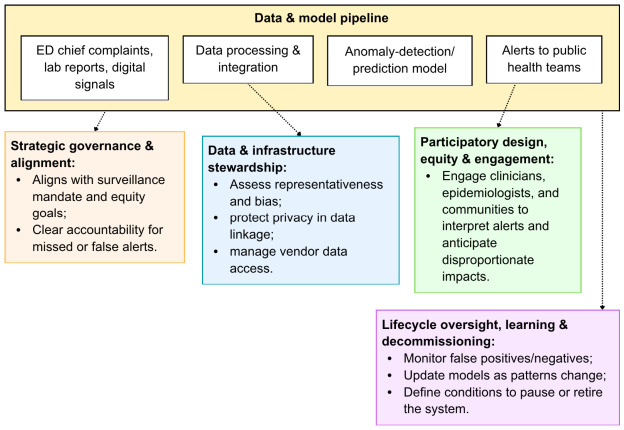
Applying the Public Health Responsible AI Capability (PH-RAIC) framework to an AI-enhanced syndromic surveillance system. The figure highlights how decisions at each stage of the AI lifecycle interact with multiple PH-RAIC domains, underscoring that governance cannot be confined to isolated checkpoints.

Figure 2 illustrates the application of PH-RAIC framework to an AI-enhanced syndromic surveillance system. Data from emergency departments, laboratories, and other sources feed an anomaly-detection model that generates alerts for public health action. The four PH-RAIC capability domains—strategic governance and alignment; data and infrastructure stewardship; participatory design, equity, and public engagement; and lifecycle oversight, learning, and decommissioning—provide checkpoints to ensure that the system aligns with public health mandates, protects privacy, promotes equity, and is continuously monitored over its lifecycle.

In practice, resource constraints may limit the extent of validation and monitoring that agencies can perform, and conflicting stakeholder priorities (e.g., between rapid detection and minimising false alarms) may make it difficult to satisfy all PH-RAIC domains simultaneously. The framework is therefore not a checklist for perfection, but a way to surface and negotiate trade-offs.

#### 3.5.2. Scenario 2: Generative AI for Public Health Communication and Disinformation Response

A national public health institute experiments with generative AI to draft culturally tailored health messages and to monitor online health disinformation.

- Strategic governance: The institute must determine how AI-generated content fits within existing communication protocols and ensure that final messaging remains under human editorial control.
- Data and infrastructure stewardship: Involves ensuring that training data for generative models does not encode harmful stereotypes and that the environmental impact of large-scale text generation is considered, especially if models are hosted in energy-intensive data centers.
- Participatory design: Would include co-creation of messages with community organizations to avoid tone-deaf content and to ensure that AI outputs respect local languages and cultural nuances.
- Lifecycle oversight: Crucial—the institute must establish rapid mechanisms for identifying and correcting AI-generated misinformation, tracking the spread of synthetic health content, and coordinating with platforms and civil society to mitigate harm [13].

**Figure 3:**
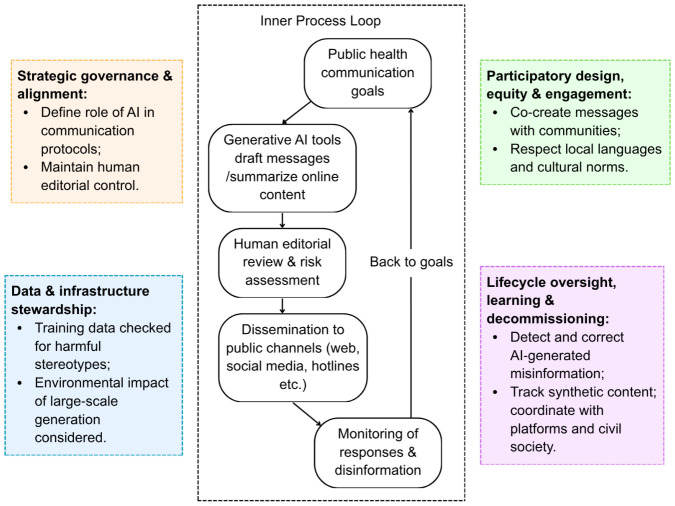
Applying PH-RAIC to generative AI for communication and disinformation.

Figure 3 shows the application of the PH-RAIC Framework to generative artificial intelligence for public health communication and disinformation response. Generative AI tools are used to draft tailored messages and to monitor and summarize online health disinformation, but all outputs pass through human review and editorial control before publication. PH-RAIC domains function as guardrails, ensuring that use of generative AI is mission-aligned, supported by robust data and infrastructure stewardship, co-designed with affected communities, and subject to ongoing lifecycle oversight to detect and mitigate harmful or misleading outputs.

### 3.6. Illustrative Mapping of Documented Implementations

To strengthen practical relevance without claiming new empirical evaluation, Table 2 maps selected examples reported in the literature and public health sources to the PH-RAIC capability domains. The mapping is illustrative rather than exhaustive, and is intended to show how PH-RAIC can be used as a structured template for identifying governance gaps, equity risks, and stewardship priorities when health data are treated as a global good. The diversity of examples in Table 2 reflects the heterogeneity of public health AI applications and underscores that PH-RAIC is intended as a flexible lens rather than a definitive taxonomy.

### 3.7. Preliminary Expert Validation of PH-RAIC

Content Validity Index values exceeded the pre-specified 0.80 threshold for all four capability domains (Table 3). Strategic governance and alignment achieved a domain CVI of 0.89 (practice-level range: 0.78–1.00); data and infrastructure stewardship achieved 0.87 (range: 0.78–1.00); participatory design, equity, and public engagement achieved 0.86 (range: 0.78–0.89); and lifecycle oversight, learning, and decommissioning achieved 0.91 (range: 0.89–1.00). At the practice level, all CVR values met or exceeded the Lawshe [21] critical value of 0.78 for N = 9.

Qualitative feedback from the panel highlighted several cross-cutting themes. First, experts affirmed that the four-domain structure comprehensively captured the major areas of organizational capability required for responsible AI in public health, with no experts recommending removal of any domain. Second, experts recommended that the distinction between PH-RAIC as a meso-level organizational framework and existing national-level readiness tools (such as the PAHO toolkit and WHO guidance) be made more explicit; this recommendation is addressed in Section 4.3 and reflected in the revised comparison Table 4. Third, several experts suggested elaborating practical guidance for under-resourced agencies, reflected in the enhanced discussion of tiered approaches in Section 3.4.3 and throughout Section 4.3. Fourth, experts from low- and middle-income country (LMIC) contexts noted that foundational data governance capabilities may need to be prioritized before more advanced lifecycle oversight capabilities can be meaningfully implemented; this is incorporated in the contextual diversity discussion in Section 4.3.

The expert panel established face validity and content validity of PH-RAIC’s conceptual structure. This represents an important first step toward empirical validation, but it does not constitute operational testing of the framework in real-world public health settings, which remains a priority for future research (see Section 4.4).

## 4. Discussion

### 4.1. Environmental and Health Externalities of AI in Public Health

Recent discussions in public health have highlighted the need to account for the environmental and health-related costs of energy-intensive AI utilization, including how these costs are distributed across communities [40]. Although empirical data specific to public health agencies remain limited, evidence from broader AI deployment indicates that large models can have substantial energy and water footprints, with associated burdens often concentrated in regions that host data centers or other energy-intensive infrastructure. Public health agencies should therefore:

- Incorporate “environmental impact” into procurement and deployment decisions, favoring efficient models, shared infrastructure, or smaller task-specific models where possible.
- Consider “cumulative burdens” on communities already exposed to environmental hazards- for example, regions hosting data centers, mines, or power plants that support AI supply chains.
- Collaborate with regulators and environmental health experts to define “metrics and thresholds” for acceptable environmental impacts of public health AI systems, and to ensure that benefits and burdens are distributed fairly.

Embedding these considerations within the data and infrastructure stewardship domain of PH-RAIC positions environmental impacts as issues of public health and equity, rather than treating them solely as technical or information technology concerns.

### 4.2. Implications for Practitioner Development and Education

There is a growing recognition that governing and regulatory authorities of public health have a central role in preparing future professionals to work responsibly with AI. In this context, PH-RAIC identifies several implications for workforce development.

- Interdisciplinary training: Curricula should combine epidemiology, biostatistics, informatics, ethics, and governance, enabling public health practitioners to critically appraise AI tools rather than treating them as opaque products.
- Organizational learning frameworks: Lessons learnt from AI deployments (successful or unsuccessful) should be systematically captured and shared across programs and jurisdictions, supporting continuous improvement of capabilities in all four PH-RAIC domains.
- New professional roles: Agencies may benefit from designated public health AI stewards or algorithmic auditors who bridge technical teams, leadership, and communities.

These developments resonate with prior calls for digital health literacy and socio-technical competence in healthcare settings, but must now adapt to the specific responsibilities and constraints of public health agencies [37].

### 4.3. Positioning PH-RAIC Within the Evolving Responsible AI Ecosystem

PH-RAIC is not intended to replace existing global principles or sector-specific frameworks. Rather, it operationalizes them for public health agencies by:

- Translating high-level values (e.g., fairness, transparency) into capability domains and concrete practices that can be assessed and strengthened using existing readiness tools and governance assessments [1,2].
- Extending lifecycle-based responsible AI approaches from individual systems to organizational portfolios and population-level interventions [18,20].
- Highlighting environmental and disinformation externalities that may be under-addressed in clinical or commercial AI frameworks but are central to public health [13].

The framework also acknowledges contextual diversity. Public health agencies in low- and middle-income countries, for example, may face severe resource constraints, limited digital infrastructure, and different regulatory environments. For these settings, PH-RAIC can guide prioritization, for instance, focusing first on foundational data governance and equity-oriented engagement, while planning for more advanced lifecycle oversight as capacity grows.

With respect to country-level application, PH-RAIC is not proposed as a country-specific or universally prescriptive model. Rather, it is intended as an adaptable capability framework that national and subnational public health agencies can contextualize according to their digital maturity, regulatory environment, resources, and public health priorities. In countries with mature digital infrastructures, it may support portfolio-level AI governance, procurement, transparency, and lifecycle monitoring. In settings where digital health systems are still developing, it may first help agencies prioritize foundational capabilities such as data quality, privacy protection, accountability, and community engagement. Therefore, PH-RAIC can be applied across country contexts, but only through local validation with public health authorities, practitioners, regulators, and affected communities.

To make this positioning explicit, Table 4 compares PH-RAIC with five leading AI governance and readiness frameworks: the WHO Ethics and Governance of AI for Health guidance [13,14], the PAHO AI Readiness Assessment Toolkit [2], the OECD AI Principles [34], the NIST Artificial Intelligence Risk Management Framework (AI RMF) [39], and the TAFES lifecycle framework [18] from which PH-RAIC’s lifecycle orientation is adapted. The comparison illustrates that PH-RAIC is distinctive in its focus on the meso-organizational level, its explicit treatment of participatory design and equity as a core domain, its integration of environmental and disinformation externalities, and its provision of illustrative measurement indicators (Table 3). It is not designed to replace any of these frameworks but to operationalize and extend them for public health agency contexts.

### 4.4. Limitations

Several limitations warrant consideration. First, we employed a targeted narrative synthesis with purposive selection of documents rather than a systematic review. As a result, our evidence base is necessarily selective and may omit relevant implementations or governance frameworks. Second, while PH-RAIC has undergone a preliminary expert panel validation establishing face validity and content validity (CVI ≥ 0.85 for all domains), it has not yet undergone empirical validation through case studies, surveys of agencies, or pilot applications in real-world public health settings. The expert panel consultation and illustrative scenarios demonstrate conceptual plausibility and expert endorsement but do not constitute formal testing in operational contexts. Third, we focus on domains of public health practice for which AI use is relatively well documented (e.g., syndromic surveillance, SDOH analytics, resource allocation, communication), and do not attempt to comprehensively cover all public health functions. The framework may require adaptation for areas such as occupational health, environmental monitoring, or mental health promotion. Fourth, while PH-RAIC identifies capability domains, example practices, and illustrative measurement indicators (Table 3), standardized maturity metrics and empirically grounded thresholds must be co-developed with practitioners, regulators, and communities through future validation work.

## 5. Conclusions

AI is reshaping the possibilities and risks of public health practice. While global principles and national strategies provide important direction, public health agencies still need practical, organization-level guidance for integrating AI efficiently, effectively, and ethically at the population level.

This paper proposes the Public Health Responsible AI Capability Framework (PH-RAIC) as a conceptual step toward that goal. By articulating four interdependent domains—strategic governance and alignment, data and infrastructure stewardship, participatory design, equity, and public engagement, and lifecycle oversight, learning, and decommissioning—PH-RAIC translates values such as transparency, accountability, fairness, ethics, and safety into capabilities that can be cultivated, assessed, and improved over time. A preliminary expert panel consultation (*n* = 9) established face validity and content validity across all four domains (CVI ≥ 0.85), providing an initial evidence base for the framework’s conceptual structure. Illustrative measurement indicators (Table 3) and a comparative analysis against five leading frameworks (Table 4) further support its practical utility and positioning.

The framework should be understood as a conceptual, expert-validated model rather than a fully operationalized assessment tool. It is intended to provide a conceptual basis for deliberation and planning at the design and development stages of AI in public health. PH-RAIC should therefore be applied through local adaptation and validation, ensuring that AI systems are aligned with public health goals, community values, human rights, and accountable human decision-making. Future work should empirically test and refine PH-RAIC in collaboration with state, tribal, local, and national public health agencies; align it with existing readiness assessments and regulatory developments; and develop and validate practical toolkits and metrics for each capability domain. Done well, such work can help ensure that public health AI enhances analytic power and efficiency while also advancing health equity, protecting rights, and supporting sustainable, trustworthy public health systems.

## Figures and Tables

**Table 1:** PH-RAIC domains, example practices, and diagnostic questions.

PH-RAIC Domain	Exemplary Capabilities/Practices	Diagnostic Questions for Agencies
**Strategic governance & alignment**	Written AI strategy explicitly linked to public health goals (equity, prevention, resilience); defined roles and responsibilities (e.g., RACI) for AI lifecycle decisions; central inventory/portfolio of AI systems in use.	How does each AI use-case contribute to our statutory public health functions and equity goals? Who is accountable if an AI-supported decision causes harm? Do we maintain an up-to-date portfolio of all AI tools in our programs?
**Data & infrastructure stewardship**	Data quality checks for representativeness and bias; privacy-preserving data access and strong cybersecurity; policies that consider the environmental impact of computing and data centers.	Which populations are underrepresented in our training data? How do we protect privacy when linking datasets across agencies or vendors? Do we assess the environmental footprint of large-scale AI deployments?
**Participatory design, equity & engagement**	Early involvement of affected communities, civil society, and frontline staff in problem framing; equity impact assessments for new AI tools; clear public communication about AI use and avenues for recourse.	Which communities are most affected by this model’s outputs, and have we involved them in design? How might this system change existing health disparities? Can the public easily find out where and how AI is used in our programs?
**Lifecycle oversight, learning & decommissioning**	Multi-disciplinary pre-deployment review (technical, legal, ethical); continuous monitoring of performance, drift, and incidents; clear triggers and processes for suspension or retirement of AI tools.	What conditions must be met before an AI system influences real-world decisions? How do we detect and respond to failures or harmful outputs in practice? When, how, and by whom do we decide to decommission an AI system?

**Table 2:** Illustrative mapping of published public health AI implementations to PH-RAIC domains and stewardship questions.

Documented Example (Source)	Public Health Function and Data Context	Primary Health-Data Stewardship Challenge(s)	PH-RAIC Domain(s) Most Salient	Illustrative Capability Checks
AI for syndromic surveillance methods [3,10]	Outbreak detection from ED chief complaints, digital signals, and unstructured reports	Data quality, representativeness, and interoperability across facilities; false alarms vs missed events	Data & infrastructure stewardship; lifecycle oversight	Assess site/subgroup performance; document data provenance and linkage; define thresholds and escalation pathways for alerts.
National vision for AI in public health [5,14]	Portfolio-level AI integration in surveillance and response programs	Strategic alignment, workforce readiness, and governance for cross-program data use	Strategic governance & alignment; data & infrastructure stewardship	Maintain AI inventory/portfolio; assign accountability (RACI); require auditable data access and cybersecurity controls.
AI early-warning outbreak surveillance [23,24]	AI-based early-warning system using digital and event-based data	Transparency of signal sources; verification and traceability; managing misinformation/false signals	Lifecycle oversight; strategic governance	Define verification workflows; log model outputs and decisions; monitor drift during crises and update responsibly.
State/local chronic disease surveillance using EHR [25]	Population surveillance using routine care EHR data across jurisdictions	Interoperability and governance for data sharing; privacy/confidentiality constraints	Data & infrastructure stewardship; strategic governance	Establish data-sharing agreements; evaluate completeness and bias; implement privacy-preserving linkage where feasible.
ML for policy-relevant social determinants [11,26]	Linked administrative and contextual datasets to study SDOH and population risk	Risk of stigmatization and biased proxies; under-representation of marginalized communities	Participatory design, equity & engagement; data stewardship	Conduct equity impact assessment; engage affected communities in problem framing; document limitations and appropriate uses.
Decision support for equitable vaccine allocation [27]	Forecasting and allocation tools for vaccination strategies	Fairness trade-offs and legitimacy of allocation criteria; transparency to the public	Strategic governance; participatory design	Co-design equity objectives; publish rationale and recourse mechanisms; audit subgroup impacts and unintended consequences.
AI-enabled public health chatbots [28]	Citizen-facing triage and information during public health emergencies	Safety, misinformation risk, privacy of user interactions, and accessibility	Lifecycle oversight; participatory design	Mandate human oversight for high-risk advice; monitor hallucinations; ensure privacy-preserving logging and clear escalation routes.
Infodemic and disinformation monitoring frameworks [29,30]	Monitoring and response to misinformation across platforms	Data provenance, transparency, and trust; dual-use risks; community legitimacy	Participatory design; lifecycle oversight	Establish rapid response protocols; track provenance and corrections; partner with community organizations for culturally competent communication.

Abbreviations: AI = artificial intelligence; ML = machine learning; ED = emergency department; EHR = electronic health record; SDOH = social determinants of health.

**Table 3:** Example measurement indicators for PH-RAIC domains (illustrative; agencies should adapt thresholds locally).

Domain	Indicator	Measurement Approach	Illustrative Threshold/Target
**Strategic governance & alignment**	Existence of a documented AI strategy	Document audit (yes/no)	Institutional AI policy document in place and reviewed ≥ every 2 years
	RACI assignment rate	% of active AI systems with assigned governance roles	≥80% of operational AI systems
	AI portfolio completeness	% of active AI tools listed in a centralized inventory	≥90% documented in inventory
	Governance review frequency	Count of structured AI governance reviews per year	≥2 governance reviews per year
**Data & infrastructure stewardship**	Data representativeness assessment rate	% of training datasets with documented bias/representativeness analysis	≥75% of deployed models
	Privacy impact assessment completion	% of AI systems with completed Data Protection Impact Assessment (DPIA) or equivalent	100% for high-risk systems; ≥80% overall
	Environmental impact documentation	% of deployments with energy or carbon footprint assessed	≥50% documented; reported publicly for large deployments
**Participatory design, equity & engagement**	Community engagement rate	% of AI systems with documented stakeholder consultation prior to deployment	≥60% for population-level tools
	Equity impact assessment completion	% of tools with formal equity impact assessment conducted	100% for tools directly affecting service delivery
	Public disclosure rate	% of AI tools listed in a publicly accessible AI register or transparency report	≥80% of operational systems
**Lifecycle oversight, learning & decommissioning**	Pre-deployment review rate	% of AI systems with completed multi-disciplinary pre-deployment review	100% for operational/decision-influencing systems
	Performance monitoring frequency	Frequency of formal performance and drift reviews per system per year	≥4 (quarterly) for operational systems
	Incident response time	Mean time from safety or equity incident detection to documented response	<72 h for safety-critical systems
	Decommissioning protocol coverage	% of systems with documented decommissioning criteria and transition plan	≥90% of operational systems

**Table 4:** Comparison of PH-RAIC with global AI governance and readiness frameworks.

Dimension	WHO Ethics & Governance (2021/2024) [13,14]	PAHO AI Readiness Toolkit (2024) [2]	OECD AI Principles [34]	NIST AI RMF (2023) [39]	PH-RAIC (This Study)
**Governance level**	Global principles	National readiness	Intergovernmental policy	Sector-agnostic operational	Meso-organizational (agency level)
**Primary focus**	Ethics principles for AI in health	National AI readiness assessment	Trustworthy AI standards	AI risk management processes	Public health agency capability domains
**Target users**	Member states, health ministries	National health ministries	Governments and industries	Organizations across all sectors	National and subnational public health agencies
**AI lifecycle coverage**	Partial (principles-based)	Limited (readiness snapshot)	Partial (policy-level)	Comprehensive (Govern, Map, Measure, Manage)	Comprehensive (design to decommissioning)
**Equity/participation**	Principle-level commitment	Assessment indicators	Referenced as a value	Referenced; no dedicated domain	Core domain with illustrative practices
**Environmental sustainability**	Limited (LMM guidance only)	Not addressed	Not addressed	Limited (risk framing)	Addressed in data stewardship domain
**Disinformation addressed**	Yes (2024 LMM guidance)	Not addressed	Not addressed	Minimal	Yes (lifecycle oversight domain)
**Measurement indicators**	Limited	Some readiness indicators	None specified	Maturity tiers defined	Illustrative indicators provided (Table 3)
**Validation status**	WHO expert consultation	Field-tested in member states	Intergovernmental consensus	Expert-developed	Conceptual + expert panel CVI ≥ 0.85
**Portfolio orientation**	Single-system framing	System-level framing	Policy-level	Single-system framing	Explicit portfolio management emphasis

Abbreviations: CVI = Content Validity Index; LMM = large multimodal model; NIST = National Institute of Standards and Technology; OECD = Organisation for Economic Co-operation and Development; PAHO = Pan American Health Organization; WHO = World Health Organization.

## Data Availability

No new data were created or analyzed in this study. Data sharing is not applicable to this article.
